# The Cost-Effectiveness of Expanding Vaccination with a Cell-Based Influenza Vaccine to Low Risk Adults Aged 50 to 64 Years in the United Kingdom

**DOI:** 10.3390/vaccines9060598

**Published:** 2021-06-04

**Authors:** Michele A. Kohli, Michael Maschio, Joaquin F. Mould-Quevedo, Mansoor Ashraf, Michael F. Drummond, Milton C. Weinstein

**Affiliations:** 1 Quadrant Health Economics Inc., 92 Cottonwood Crescent, Cambridge, ON N1T 2J1 Canada; michael.maschio@quadrantHE.com; 2 Seqirus USA Inc., 25 Deforest Avenue, Summit, NJ 07901, USA; joaquin.mould-quevedo@seqirus.com; 3 Seqirus UK, 29 Market St., Maidenhead SL6 8AD, UK; mansoor.ashraf@seqirus.com; 4 Centre for Health Economics, University of York, Heslington, York YO10 5DD, UK; mike.drummond@york.ac.uk; 5 Harvard T.H. Chan School of Public Health, 718 Huntington Avenue, Boston, MA 02115, USA; mcw@hsph.harvard.edu

**Keywords:** influenza vaccine, cost-effectiveness, economic modeling

## Abstract

Background: In response to COVID-19, the UK National Health Service (NHS) extended influenza vaccination in 50- to 64-year-olds from at-risk only to all in this age group for the 2020/21 season. The objective of this research is to determine the cost-effectiveness of continuing to vaccinate all with a quadrivalent cell-based vaccine (QIVc) compared to returning to an at-risk only policy after the pandemic resolves. Methods: A dynamic transmission model, calibrated to match infection data from the UK, was used to estimate the clinical and economic impact of vaccination across 10 influenza seasons. The base case effectiveness of QIVc was 63.9% and the list price was GBP 9.94. Results: Vaccinating 50% of all 50- to 64-year-olds with QIVc reduced the average annual number of clinical infections (−682,000), hospitalizations (−5800) and deaths (−740) in the UK. The base case incremental cost per quality-adjusted life-year gained (ICER) of all compared to at-risk only was GBP6000 (NHS perspective). When the cost of lost productivity was considered, vaccinating all 50- to 64-year-olds with QIVc became cost-saving. Conclusion: Vaccinating all 50- to 64-year-olds with QIVc is likely to be cost-effective. The NHS should consider continuing this policy in future seasons.

## 1. Introduction

In the United Kingdom (UK), seasonal influenza vaccines have been provided annually to those in a clinical risk group as well as those aged 65 years and above since 2000 [1]. The clinical risk groups considered to be at higher risk of morbidity and mortality associated with an influenza infection include those with chronic respiratory disease, chronic heart disease, chronic kidney disease, chronic liver disease, chronic neurological disease, diabetes, immunosuppression, asplenia or dysfunction of the spleen, and morbid obesity, as well as pregnant women. While annual coverage for those 65 years and over from the 2007/08 to 2016/17 seasons has averaged 73% in England, the average coverage amongst at-risk groups excluding pregnant women has been lower at 49% [1]. In 2011, the Joint Committee on Vaccination and Immunisation (JCVI) recommended expansion of routine vaccination to healthy or low risk individuals aged 2 to 16 years because it was likely to be a cost-effective intervention [2]. Modeling predicted that routine vaccination would prevent disease in the vaccinated individuals and indirectly prevent cases in other age groups by reducing transmission [3].

For the 2020/21 season, the National Health Service (NHS) extended the influenza program to all individuals aged 50 to 64 years to support the health and care system given the co-circulation of influenza and COVID-19 [4]. The objective of this research is to determine the cost-effectiveness of continuing to provide quadrivalent influenza mammalian cell-based vaccine (QIVc) vaccinations to all individuals aged 50 to 64, including low and at-risk people, once the COVID-19 pandemic has resolved.

## 2. Methods

### 2.1. Overview

A compartmental transmission model with a Susceptible–Exposed–Infectious–Recovered (SEIR) structure was used to predict the number of influenza infections and their consequences in the population of the United Kingdom. The dynamic model and the calibration of the model inputs are described in the File S1. All individuals with symptomatic infections, which are also called clinical infections, are subdivided into cases that require no care, cases that require outpatient only care, and cases that require hospitalization. While deaths from influenza do occur in the community, it was conservatively assumed for this analysis that all deaths occur amongst hospitalized cases only.

### 2.2. Comparators

A “current coverage”, or at-risk only, scenario was constructed using vaccine uptake inputs from a previously published analysis (Table 1) [5]. This scenario was compared to each of the scenarios displayed in Table 1, in which low risk 50- to 64-year-olds are also vaccinated with QIVc at varying coverage levels. The coverage rate is uncertain, but it is expected to be less than that seen among persons 65 years and over. Therefore, several scenarios were run, assuming 40%, 50%, and 60% coverage of the low risk group. Coverage of the vaccine in the at-risk group was 48.6% in the “current coverage” scenario. This was adjusted upwards for the 50% and 60% scenarios so that coverage of the at-risk group was at least equivalent to that of the low risk group.

### 2.3. Vaccine Effectiveness

The vaccines given to each age group were chosen to reflect the JCVI-recommended and NHS-funded vaccines for the 2021/22 season [6,7,8]. In this analysis, individuals aged 18 to 64 years old receive a quadrivalent influenza cell-based vaccine (QIVc) as this is one of the vaccines that is both recommended by the JCVI and funded by the NHS for this age group. QIVc is a version of influenza vaccine that is cultured in mammalian cells in order to avoid viral adaptations that reduce the match to the recommended strains that may occur in the vaccines produced in eggs. Most data on the effectiveness of this vaccine come from the use of the vaccines in the United States, starting in the 2017/18 season [9,10,11,12,13,14]. The effectiveness of all influenza vaccines varies by season and depends in part on the degree to which the vaccine strains, which are determined annually by the World Health Organization, [15] match the strains that are circulating locally. Therefore, effectiveness may vary by season and location. For this analysis, the effectiveness of QIVc was estimated based on the latest Public Health England (PHE) data from the UK during the 2019/20 season. PHE collected data from five primary care influenza sentinel swabbing surveillance schemes to conduct a test-negative case control study of vaccine effectiveness in this season [16]. They reported the effectiveness of QIVc against all influenza types amongst 18- to 64-year-olds, adjusted for confounders, to be 63.9% (95% confidence interval of 26.9% to 82.2%). This effectiveness estimate was used for the base-case analysis, with scenario analyses conducted using the upper and lower bounds of the 95% confidence interval estimates.

The vaccines used in the age groups not targeted for this analysis were also determined based upon the 2021/22 NHS recommendations for influenza vaccines [6,7]. Children aged 6 to 23 months received quadrivalent influenza egg-culture vaccine (QIVe), while 2- to 17-year-olds received quadrivalent live-attenuated influenza vaccine (QLAIV). Individuals ages 65 years and above received adjuvanted quadrivalent inactivated vaccine (aQIV). The effectiveness of each of these vaccines is described in the File S1.

### 2.4. Costs

The analyses were conducted using both the cost perspective of the NHS and Personal Social Services, as recommended by the National Institute for Health and Care Excellence (NICE) [17], and a societal perspective that includes productivity losses. The unit cost for QIVc was assumed to be GBP 9.94, QIVe GBP 8.97, QLAIV GBP 18.00, and aQIV GBP 11.88 [18]. The unit cost for QIVe is an average of the NHS list prices for the multiple funded QIVe formulations. Vaccine administration was assigned a cost of GBP 10.06 per dose based on the directed enhanced service payments for the seasonal influenza program (2020/21) for general practitioners [19].

Many of the symptomatic influenza cases receive no medical care. As in previous UK analyses, [20,21] only 10% of infections were assumed to receive outpatient care and these were assigned the average age-specific costs displayed in Table 2. This cost from Pitman and colleagues, [22] inflated to 2020 values [23], included the cost of consultations and some medications. The cost of anti-viral treatment [24] was also added as recommended by UK guidelines [25] based on previously published UK analyses [26]. The age- and risk-group specific probability of hospitalization rates given clinical infection (Table 2) were derived using an analysis of UK data by Cromer and colleagues as described in the File S1 [27]. Costs per admission (Table 2) were also from Pitman and colleagues [22]. The case fatality rates for hospitalized cases (Table 2) were taken directly from Cromer and colleagues [27].

For the societal perspective, all NHS and PSS costs plus the cost of time lost from work, estimated using the human capital method, were included. For the base case, the average time loss per working person developing a case of influenza was assumed to be 4.0 days based on data from a study of individuals with laboratory confirmed influenza cases in France [28,29]. The proportion working (Table 2) was estimated by age group based on UK data [30]. The daily wage was estimated as GBP 114.75 based on a UK annual survey [30].

### 2.5. Utilities

A quality-adjusted life years (QALYs) decrement, or a disutility weighted by time spent in the health state, was applied to cases of influenza as in previous UK analyses. A decrement of 0.0075 [3,34] was applied to uncomplicated cases of influenza based on individuals with influenza-like illness, and 0.0180 was applied to hospitalized cases based on individuals with inpatient pneumococcal disease [3,35]. The discounted number of QALYs lost due to death from influenza was calculated using expected survival [36] and expected age-specific utility values [37]. QALYs lost due to death in the low and at-risk groups differ, as individuals in the at-risk groups have comorbidities that result a lower quality-adjusted life expectancy compared to low risk individuals in the same age group. However, because the distribution of comorbidities in the target population is unknown, it was not possible to develop separate estimates of QALYs lost to death in the at-risk and low risk groups. Instead, we conducted a sensitivity analyses where we reduced the mortality rate in low risk individuals to see if having a differential impact of mortality by risk group would impact our results.

### 2.6. Analysis

The model was run for 10 seasons, but all outcomes are presented as averages per season over the 10 years. The size of the population in Year 1 of the simulation was based on 2019 data and then assumed to change according to the average growth rate from the Office for National Statistics (Table 2) [31]. The proportions of at-risk individuals due to complications was based on data presented in previously published UK analyses (Table 2) [3,32,33]. A discount rate of 3.5% for both costs and outcomes was used in line with NICE recommendations [17]. 

As described above, scenario analyses were conducted using multiple vaccine coverage and QIVc effectiveness estimates. In addition, deterministic sensitivity analyses (DSAs) with key variables were conducted to determine their impact on the incremental cost-effectiveness ratio (ICER) (NHS cost perspective). These analyses were run assuming 50% coverage and 63.9% effectiveness of QIVc. For example, the hospitalization rates were varied from 50% to 200% of the base case values in order to represent the variation in severity of the influenza epidemic across seasons [16,38]. Varying the hospitalization rates also varies the number of deaths due to influenza that occur. PHE has estimated that the numbers of deaths attributable to influenza have varied substantially over the last 5 years [16]. The QALY decrement for uncomplicated and complicated cases was varied from 0.0002 and 0.0146, respectively, to 0.0271 and 0.0217, respectively. These ranges were based on the 95% confidence interval estimated in the source publication [3]. In addition, the QALY decrement associated with death due to influenza was considered by varying the base-case age-specific utility values by 10%. As described above, the mortality rate in low risk individuals was reduced by 50% to determine if having a differential QALY loss with death by risk group impacts the results. Finally, the discount rate for costs and outcomes was also reduced to 1.5%, as in its recent methods review NICE has acknowledged that ‘the evidence suggests there is a case to change the reference discount rate to 1.5% per year for both costs and effects’ [39]. 

For the societal perspective, the amount of time lost from work was varied in DSAs. Time lost from work due to influenza was varied from 1.2 to 6.8 days based on the standard deviation of time loss from work in Carrat et al. [29] In addition, scenario analyses assuming that all working individuals need to take 1 h off work in order to be vaccinated (thereby adding to the cost of vaccine administration) were conducted.

Probabilistic sensitivity analyses were conducted by varying the inputs to the economic model—including percent of patients seeking a medical care visit, inpatient complications, outpatient complications, influenza-related mortality rates, QALYs lost for infections with inpatient complications, and QALYs lost for infections with no complications—using the distributions described in the File S1.

## 3. Results

### 3.1. Base Case Analysis

When vaccination with QIVc is extended from an at-risk only strategy to all individuals aged 50 to 64 years, the morbidity and mortality associated with influenza decreases. As shown in Table 3, the impact depends upon vaccine coverage, but, in general, the numbers of clinical infections, hospitalizations and deaths decrease as the number of vaccinations of individuals aged 50 to 64 years increases. While there is a direct benefit to the 50- to 64-year-olds themselves, the model predicts that more morbidity and mortality is prevented in the other age groups through a reduction in transmission. For example, with 50% coverage, a reduction of about 1200 hospitalizations annually is seen in the 50- to 64-year-olds, while a reduction in 4600 hospitalizations is seen in other age groups. The results by age group are presented in the File S1 (Table S6).

Increasing the number of vaccinations results in a net cost to the NHS due to the increased cost of the vaccine itself and vaccine administration, but, as shown in Table 4, some of this cost is offset by reduction in treatment costs associated with infection. With 50% coverage, the expected discounted cost of influenza (including vaccinations and treatment of influenza cases) to the NHS over 10 seasons is approximately GBP 4.8 billion or an average of GBP 480 million per year. This is more than the expected discounted cost of the current program to the NHS—GBP 4.1 billion over 10 seasons or GBP 410 million per year. However, by preventing illness and deaths, 6446 and 4483 discounted QALYS will be gained, respectively, per year, for an annual total of 10,929 discounted QALYs gained. With 50% coverage of 50- to 64-year-olds, the average annual incremental NHS discounted cost compared to the current strategy is GBP 66,000,000. The incremental cost per QALY gained of vaccinating 50% of all 50- to 64-year-olds is therefore approximately GBP 6000 (Table 5). While Table 4 shows that varying vaccine coverage between 40% and 60% does impact costs and expected QALYs, the incremental cost-effectiveness ratio (ICER) of vaccinating low risk 50- to 64-year-olds remains at approximately GBP 6000 per QALY gained irrespective of coverage within this range (Table 5). The results by age group are presented in the File S1 (Table S7).

When productivity costs are introduced into the analysis, each case of influenza prevented leads to more societal savings which more than offset the NHS costs of vaccination. The total societal costs decrease as the coverage of the 50- to 64-year-olds increases (Table 4). For the base case, when considering a societal perspective, vaccinating the low risk group provides both cost savings, due to productivity losses averted, and more QALYs regardless of the coverage level and is therefore considered to be dominant (Table 5).

**Figure 1:**
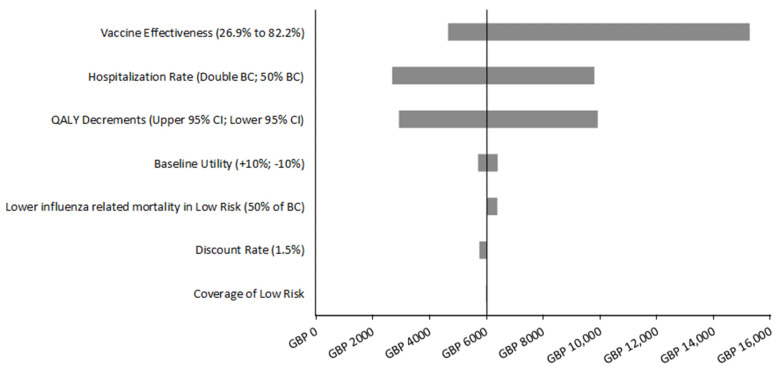
Tornado diagram: impact of the deterministic sensitivity analyses on the incremental cost per quality-adjusted life-year (NHS cost perspective) associated with vaccinating 50% of low risk individuals ages 50 to 64 years old.

The DSAs summarized in the Tornado diagram in Figure 1 demonstrate that the ICER (NHS perspective) is most sensitive to the effectiveness of the vaccine, as expected. If the effectiveness of QIVc is as low as 26.9%, then the cost per QALY gained of vaccinating the low risk group increases to GBP 15,000, but it may be as low as GBP 4700 if the vaccine is 82.2% effective. The results are next most sensitive to fluctuations in hospitalization rates, which impact both the cost of influenza treatment and the number of deaths because all deaths are assumed to come from hospitalized cases. When the rate of hospitalization is half of what was assumed in the base case, the number of hospitalizations and deaths prevented annually decrease from 5807 and 737 in the base case to 2903 and 368, respectively. When the rate is doubled, the number of hospitalizations prevented increases to 11,614, while the number of deaths prevented increases to 1474. These changes in influenza outcomes cause the ICER to vary from GBP 2700 (high hospitalization) to GBP 9800 (low hospitalization). Next, the ICER is most sensitive to variation in the QALY decrements associated with influenza cases: the ICER may range from GBP 2900 to GBP 9900 depending on the value used within the range considered. Varying the discount rates, the coverage of the low risk group, the baseline utility associated with the population, and changing the mortality in the low risk group only have a minimal impact on the ICER.

The DSAs demonstrate that the productivity costs that are saved for the 50% vaccination coverage of 50- to 64-year-olds scenario, depend upon the average number of days lost from work per case. If an average of 4 days is lost from work per case, there is a net saving in total societal costs when implementing the strategy, regardless of whether time lost from work due to seeking vaccination is assumed to be 0 or 1 h. If only 1.2 days are lost, the gains in productivity do not offset the cost of vaccination, but implementation of the low risk strategy is still cost-effective. From the societal perspective, the ICER is GBP 1900 per QALY gained assuming 0 h of work time loss for vaccination and GBP 7200 assuming 1 h. The detailed results from these sensitivity analyses are found in the File S1 (Table S8).

### 3.2. Probabilistic Sensitivity Analysis

A probabilistic sensitivity analysis was conducted using the base case effectiveness of 63.9% and also for the lower and upper 95% confidence interval values of 26.9% and 82.2%. As shown in the File S1 (Figure S5), in 100% of the simulations, the ICER is below the willingness-to-pay threshold of GBP 25,000 with an absolute vaccine effectiveness of 26.9%, below GBP 10,000 for an effectiveness of 63.9% and below GBP 8000 for an effectiveness for an effectiveness of 82.2%.

## 4. Discussion

Maintaining the 2020/21 influenza season policy of vaccinating low risk 50- to 64- year-olds is expected to decrease morbidity and mortality in the UK population and be a cost-effective policy during a typical influenza season using QIVc. The total cost to the NHS and the overall benefits will depend on the coverage achieved in this population, but regardless, the ICER of vaccinating low risk 50- to 64-year-olds compared to not vaccinating this sub-group is around GBP 6000 per QALY under base case assumptions. When the cost of lost productivity is considered, the policy has the potential to be both more effective and cost-saving from the societal perspective. Overall, our analysis indicates that maintaining a policy of vaccinating all 50- to 64-year-olds in the UK post-pandemic is cost-effective. 

The burden of influenza illness varies substantially from season to season. For example, PHE estimates that the number of deaths attributed to influenza in England at 3966 in the 2018/19 season and 22,087 in the 2017/18 season. There may be multiple reasons for this variation, including vaccine effectiveness against the local circulating strains and the virulence of the circulating strains. We conducted sensitivity analyses varying vaccine effectiveness. The range used for vaccine effectiveness in sensitivity analyses was based on the PHE test-negative design study, which is the preferred design for observational studies of influenza vaccine effectiveness [40]. As recommended by Newell et al., [41] this study uses the outcome of laboratory confirmed influenza and we assume that this effectiveness can be applied equally to prevention of transmission, hospitalization and deaths. Similar to many studies using laboratory confirmed influenza infections as the outcome of interest, the 95% confidence interval from this study was wider than in the database analyses focusing on medical encounters [42]. However, even when we used this wide range in our DSAs, the ICER ranged from GBP 4700 to GBP 15,000 indicating that this policy is likely to be cost-effective assuming a willingness-to-pay threshold of GBP 20,000 or greater. 

We also conducted DSAs with variables that would reflect variation in influenza virulence including hospitalization rates, case fatality, QALY decrement and time lost from work. All of the variables had less of an impact on the ICER than varying the vaccine effectiveness. Furthermore, in the PSAs for the various vaccine effectiveness levels, where the variables representing the impact of influenza were systematically varied, the ICER associated with implementing the strategy remained below GBP 25,000.

Assuming 50% coverage, offering the vaccine to the entire 50- to 64-year-old population, including those at low risk, would require approximately 5,300,000 additional vaccinations annually. If vaccination is restricted to those age 55 to 64 years only, the number of additional vaccinations required drops to 3,400,000. For the 60 to 64 age group, it decreases to 1,700,000. These age-varying strategies have almost identical ICERs (in technical terms, they lie on a nearly linear section of the efficiency frontier) so the choice among them might well be budget-driven.

### 4.1. Comparison to Previous Studies

In 2006, Turner et al. [43] published a cost-effectiveness analysis examining this issue of extending immunization with a conventional egg-based trivalent influenza vaccine in the UK to healthy adults aged 50–64 years. A static model was used, and therefore the estimates did not include any of the indirect benefit (herd immunity) associated with reducing the number of susceptible individuals in the population. The efficacy of the vaccine in this age group was 69% in base case and varied from 55% to 79% in sensitivity analyses. The cost of the vaccine was lower at GBP 7.24, but the costs associated with health care treatment of influenza and time loss were also lower as they were in 2002 UK prices. The base case ICER (NHS cost perspective) in that study was GBP 6174 per QALY gained. The ICER using the societal cost perspective was higher at GBP 10,766 because they assumed a 1-h time loss cost for the working individuals to receive an influenza vaccine for their base case. Given the widespread influenza vaccination settings that are now available in the UK, we assumed in the base case that individuals did not need to lose time from work in order to be vaccinated. For the societal perspective, Turner et al. used a lower estimate of average days lost per case of influenza (2.9 days) but a similar range to ours for sensitivity analyses.

Newall et al. [41] reviewed multiple analyses that had been conducted using static models looking at this research question in various countries and concluded that vaccination of all 50 to 64 years old is likely to be cost-effective. The results were most impacted by the estimates of vaccine effectiveness and of the incidence of the serious outcomes associated with influenza.

In 2015, Baguelin et al. [3] considered the issue of expanding vaccination to healthy individuals in various age groups, including the 50- to 64-year-olds, using a dynamic model. Their analysis, like ours, therefore included benefits of reducing transmission to other groups in society. They assumed a vaccine effectiveness of 70% for seasons in which vaccines strains were well-matched to the circulating strain and 42% for poorly matched seasons. Vaccine cost, including administration, was GBP 15.85. They estimated that the ICER (NHS cost perspective) associated with expanding vaccination to healthy 50 to 64 years old on top of vaccinating as per what we refer to as the “current strategy”, was GBP 8093 per QALY.

### 4.2. Limitations

As with any analysis based on modelling, there are a number of limitations associated with this model. Influenza seasons are highly variable and our model is calibrated to match only two rates of infections. As Newell et al. [41] commented, the severity of influenza seasons was one of the important determinants of cost-effectiveness in past publications analyzing vaccination of those aged 50 to 64 years of age. Influenza seasons are highly variable and the dynamic model used in this analysis contained two different calibrated seasons: one that was primarily type A and one that was a mix of types A and B. We did not otherwise vary the number of clinical influenza infections predicted by the model, but we did vary the proportions of hospitalizations and deaths associated with those infections in sensitivity analyses. In addition, our model is conservative as we predict 2834 deaths in the UK in all age groups in an average season. Had we included estimates of mortality due to influenza cases that are not hospitalized, this number would have been higher. PHE has estimated that the number of influenza-attributable deaths have been at least 3966 per season in England alone over the past five seasons [16]. As we demonstrated in the sensitivity analyses, whenever the severity of influenza is increased, the cost-effectiveness vaccinating all 50- to 64-year-olds increases. Therefore, our analysis may underestimate the value associated with maintaining this policy.

While the model includes the most recent data available, several model inputs such as contact matrices, influenza hospitalization rates and case fatality rates were based on studies conducted prior to 2010. If influenza burden has changed over the last decade, the model will not capture these variations in hospitalization or case fatality rates. Furthermore, it is unclear whether the COVID-19 pandemic will impact the burden of influenza in the future. It is possible, for example, that social distancing practices during the pandemic will lead to post-pandemic changes in behavior that impact contact rates between individuals. The mass vaccination campaign associated with COVID-19 may change adults’ willingness to receive an annual influenza vaccine. Ultimately, any such long-term changes would impact the transmission of influenza in the UK population. However, the direction of such changes is still unclear. 

A systematic review of studies of the impact of influenza on productivity reported a large degree of heterogeneity in study methods and quality [28]. They reported that mean time lost form work in studies of laboratory confirmed influenza varied from 1.5 to 4.9 days lost. In a more recent study of a community cohort of individuals in the UK over severe seasons, 34% of the 62 employed individuals aged 16 years and above who had PCR-confirmed influenza A reported taking an average of 4 days off work due to their illness [44]. This implies an average of 1.4 working days lost for all of those employed. The authors suggest that the estimate of days lost may be lower than reported in other studies because they include milder cases that do not seek medical care. While this value is lower than our base case value, it does fall within the range of the sensitivity analyses that we conducted. We did not include caregiver time loss from work, as there are limited data focusing on cases in children only [28]. This is a conservative approach because including this cost would also add to the overall burden of lost productivity associated with influenza [45]. Overall, for the age group under consideration, the potential savings in productivity costs associated with vaccination of this age group is important from a societal perspective as the majority of individuals are working.

Finally, there is a lack of data on the impact of influenza on quality of life, with several studies using estimates from studies of pneumonia patients. In the same study of community-based influenza described above, Fragaszy et al. [44] also measured utility and their findings suggest that the quality-of-life decrement may also be less than used in past cost-effectiveness analyses. On the other hand, the impact on hospitalization and serious respiratory disease on elderly individuals following hospital discharge may be underestimated [46]. In our sensitivity analyses, we included a wide range of quality-of-life decrements and still found vaccination of the low risk individuals to be cost-effective.

## 5. Conclusions

The UK government has decided to extend vaccination in order to protect the capacity of the NHS due to the COVID-19 pandemic. Our analysis demonstrates that when considering influenza in isolation, continuing to vaccinate these individuals with QIVc in future seasons is likely to be cost-effective from the perspective of the NHS and provide cost-savings when lost productivity is considered. UK policy makers should consider continuing this policy in future seasons once the concern over COVID-19 pandemic is mitigated.

## Figures and Tables

**Table 1:** Vaccine coverage for the strategies compared in the analysis.

Age Group	Current Strategy (Reference)		40% (50 to 64 Years)		50% (50 to 64 Years)		60% (50 to 64 Years)	
	Low Risk	At-Risk	Low Risk	At-Risk	Low Risk	At-Risk	Low Risk	At-Risk
**6–23 months**	0.10%	3.10%	*	*	*	*	*	*
**2–6 years**	28.10%	48.60%	*	*	*	*	*	*
**7–17 years**	27.60%	48.60%	*	*	*	*	*	*
**18–49 years**	0.00%	48.60%	*	*	*	*	*	*
**50–54 years**	0.00%	48.60%	40%	*	50%	50%	60%	60%
**55–59 years**	0.00%	48.60%	40%	*	50%	50%	60%	60%
**60–64 years**	0.00%	48.60%	40%	*	50%	50%	60%	60%
**65–74 years**	68.00%	68.00%	*	*	*	*	*	*
**75 years and above**	80.00%	80.00%	*	*	*	*	*	*

* Same as the current or reference strategy.

**Table 2:** Key population and economic model inputs.

Age Group	Population Inputs			Probability of Hospitalization Given Symptomatic Infection		Case Fatality Rate (per 1000 Hospitalized Cases)		Cost of Outpatient Care		Cost of Hospital Admission (GBP)	Proportion Working by Age Group
	Population in Year 1	Annual Rate of Growth	Proportion at High Risk (At-Risk)	Low Risk	At-Risk	Low risk	At-Risk	Low Risk (GBP)	At-Risk (GBP)		
**6–23 months**	1,551,398	−0.2%	4.9%	3.59%	3.16%	0.43	17.45	GBPGBP94.35	GBP98.36	GBP1985.33	0%
**2–6 years**	4,009,154	−0.2%	7.3%	2.72%	3.46%	0.43	17.45	GBPGBP74.73	GBP80.74	GBP1985.33	0%
**7–17 years**	8,621,803	0.6%	9.6%	0.16%	1.03%	0.74	24.43	GBP76.24	GBP84.25	GBP2006.59	3.92%
**18–49 years**	27,477,085	0.1%	9.1%	0.19%	1.18%	6.07	39.97	GBP104.07	GBP106.55	GBP2053.65	80.54%
**50–59 years**	9,062,377	−0.8%	18.3%	0.54%	3.25%	6.07	39.97	GBP124.51	GBP126.99	GBP2451.38	72.40%
**60–64 years**	3,757,250	2.1%	18.3%	0.60%	3.61%	6.07	39.97	GBP124.51	GBP126.99	GBP2451.38	72.40%
**65–74 years**	6,694,529	1.1%	45.0%	3.12%	5.69%	185.29	428.52	GBP125.35	GBP125.35	GBP6618.61	10.80%
**75 years and above**	5,693,825	2.9%	45.0%	3.15%	5.75%	185.29	428.52	GBP125.35	GBP125.35	GBP6618.61	10.80%
**Source**	[31]	[31]	[3,32,33]	[27]	[27]	[27]	[27]	[22,24,25]	[22,24,25]	[22]	[30]

**Table 3:** Base case results: the average annual change in clinical cases when low risk individuals ages 50 to 64 years old are vaccinated, by coverage level.

	Number with Current Strategy (0% Low Risk)	Change with 40% (50 to 64 Years)	Change with 50% (50 to 64 Years)	Change with 60% (50 to 64 Years)
Vaccinations	16,140,518	4,198,394	5,280,907	6,565,605
Clinical Infection	2,615,577	−544,718	−682,036	−844,277
Hospitalizations	22,148	−4629	−5807	−7257
Deaths	2834	−588	−737	−916

**Table 4:** Base case results: the average annual discounted costs and quality-adjusted life-years with different level of coverage of low risk individuals aged 50 to 64 years old.

	Current Strategy (0% Coverage of Low Risk)	40% Coverage of Low Risk	50% Coverage of Low Risk	60% Coverage of Low Risk
Cost of Vaccinations	GBP 180,483,831	GBP 216,398,306	GBP 225,658,483	GBP 236,648,224
Cost of Vaccine Administration	GBP 139,240,897	GBP 175,588,948	GBP 184,960,917	GBP 196,083,331
Cost of Medical Care Visits	GBP 22,781,219	GBP 17,851,296	GBP 16,620,253	GBP 15,172,047
Cost of Hospitalizations	GBP 71,397,019	GBP 56,329,214	GBP 52,517,054	GBP 47,893,573
**Total NHS Costs**	**GBP 413,902,966**	**GBP 466,167,764**	**GBP 479,756,707**	**GBP 495,797,175**
Productivity	GBP 538,666,193	GBP 418,970,603	GBP 389,271,255	GBP 354,455,465
**Total Societal Costs**	**GBP 952,569,159**	**GBP 885,138,367**	**GBP 869,027,961**	**GBP 850,252,641**
QALYs	51,414,486	51,423,206	51,425,415	51,428,069

NHS—National Health Service; QALY—Quality-adjusted life-years.

**Table 5:** The incremental cost per quality-adjusted life-year ratio associated with vaccinating low risk individuals ages 50- to 64-year-olds across various coverage and vaccine effectiveness levels.

	Incremental Cost per QALY Gained Compared to the Current Strategy		
Vaccine Effectiveness	40% (50 to 64 Years) vs. Current	50% (50 to 64 Years) vs. Current	60% (50 to 64 Years) vs. Current
**NHS Perspective**			
26.90%	GBP 15,431	GBP 15,268	GBP 14,975
63.90%	GBP 5993	GBP 6025	GBP 6029
82.20%	GBP 4574	GBP 4661	GBP 4745
**Societal Perspective**			
26.90%	GBP 1642	GBP 1523	GBP 1337
63.90%	Dominates *	Dominates *	Dominates *
82.20%	Dominates *	Dominates *	Dominates *

* Dominates means that increased coverage of the low risk group is more effective and cost savings compared to no coverage. NHS—National Health Service; QALY—Quality-adjusted life-years; vs.—versus.

## Data Availability

Not applicable.

## Supplementary Materials

The following are available online at https://www.mdpi.com/article/10.3390/vaccines9060598/s1, File S1: Cost-effectiveness of Expanding Vaccination with a Cell-Based Influenza Vaccine to Low-Risk Adults Aged 50 to 64 Years in the United Kingdom.
