# Supplementary material for: The Cost-Effectiveness of Expanding Vaccination with a Cell-Based Influenza Vaccine to Low Risk Adults Aged 50 to 64 Years in the United Kingdom
Source: Vaccines (Basel). 2021 Jun 4;9(6):598. doi: 10.3390/vaccines9060598 (PMC8228189; doi:10.3390/vaccines9060598)
Supplement: Supplementary file 1 [file vaccines-09-00598-s001.zip › vaccines-1214550-supplementary.pdf]

Cost-effectiveness of Expanding Vaccination with a Cell-Based Influenza Vaccine to Low-Risk Adults Aged 50 to 64 Years in the United Kingdom

Supplementary Materials

Table of Contents

1. Dynamic model structure 2
2. Mathematical Equations for the Dynamic Model 5
3. Dynamic Model Calibration 6
4. Calibration of Hospitalization Rates 11
5. Vaccine Effectiveness 11
6. Probabilistic Sensitivity Analyses 13
7. Additional Results Tables and Figures 16
8. References 20

List of Tables

Table S1. Characteristics of the distribution of age-specific susceptibility distribution used in the calibration.. 8

Table S2. Final dynamic model inputs, by scenario.. 9

Table S3. Characteristics of the 10 influenza seasons used in the analysis 12

Table S4. Absolute effectiveness inputs for QIVe and QLAIV by age and viral type.. 12

Table S5. Probabilistic sensitivity analysis base case values and standard errors 13

Table S6. Base case results: the average annual change in clinical cases when low-risk individuals ages 50 to 64 years old are vaccinated, by coverage level and age group. 16

Table S7. Base case results: the average annual discounted costs and quality-adjusted life-years with different level of coverage of low-risk individuals aged 50 to 64 years old, by age group. 17

Table S8. The impact of days lost per case of influenza on productivity costs and the societal costs per quality adjusted life-year gained. 18

List of Figures

Figure S1. Transmission model structure 3

Figure S2. Resource use model structure 4

Figure S3. Incidence of clinical infection in an unvaccinated population (A only scenario) 10

Figure S4. Incidence of clinical infection in an unvaccinated population (Low A / High B Scenario).. 10

Figure S5. The cost-effectiveness acceptability curves for probabilistic sensitivity analyses with vaccine effectiveness set to 26.9% (Panel A), 63.9% (Panel B), and 82.2% (Panel C). 19

1. Dynamic model structure

The compartmental transmission model structure is shown in Figure S1 below. Each of the compartments is divided into 15 age groups and by risk of complication from infection (low or at-risk). All individuals the simulation begin in the susceptible, unvaccinated compartment at the start of the influenza season, except for a few individuals representing the imported influenza that is required to start the epidemic. As in a standard SEIR (Susceptible, Exposed, Infected, Resistant) model, individuals move from susceptible to exposed according to the force of infection, which is a function of the rates of effective contacts between susceptible and unsusceptible individuals in the population. The mathematical equations for this model are given in the next section. As with other transmission models,[1-4] the rate of effective contact is a function of the age-specific contact matrix[5] and the transmissibility of the virus per contact. Unique to this approach, as each season is treated independently, a proportion of the population in the susceptible compartment may actually have naturally acquired immunity from infection in the past season.

Following an effective contact, individuals develop a latent infection that cannot be transmitted and transition to the exposed compartment. Next, they move into the infection compartment where they can transmit their infection until entering the recovered compartment. As in Baguelin 2012[6], there are two exposed and two infected compartments (not shown in Figure S1) which allow the latent and infectious periods to be gamma-distributed. The rate of loss of latency is a function of the average latent period. Similarly, the loss of infectiousness is a function of the average duration of an influenza infection. Following recovery from an infection, individuals move into a recovered compartment and remain there until the end of the influenza season. At the start of a new season, the simulation is reset and all individuals are placed in the susceptible compartment again.

All model compartments are also stratified between low risk and at-risk individuals due to complications from influenza (also not shown on Figure S1). Probability of transitioning between compartments is the same for low and at-risk individuals, and random mixing between the groups is assumed. These populations are separated to track differential vaccine coverage as well as differential risk of hospitalization and death following infection.

Influenza A is modelled independently from influenza B, using the same model structure. There is no vaccine cross-protection of A and B. Vaccine effectiveness is a function of the proportion of A/H1N1 and A/H3N2 infections and effectiveness against each A type. For type B infections, an overall effectiveness was applied to both Yamagata and Victoria lineages. Vaccine-mediated protection is assumed to last for 1 year (i.e., the entire influenza season). Vaccination is assumed

to occur at a constant rate between week 40 of the current year and week 5 of the following year (end of January). Individuals in all unvaccinated compartments are eligible, as a portion of the infections would have occurred without development of clinical symptoms such that infection status may not be known by the individual. Effective vaccination is assumed to fully protect a portion the vaccinated individuals, while those unsuccessfully vaccination remain susceptible.

The time step of the simulation was set to be 0.1 days based on a range of epidemiological systems explored by Keeling and colleagues.[7] The model was programmed in Microsoft Excel.

Only a portion of the individuals who are infected develop clinical symptoms while the remainder are asymptomatic. The number of infections with clinical symptoms for a season are output into the resource use decision tree shown in Figure S2 of the manuscript in eight different age groups, each stratified by risk of complications from influenza (at-risk or low risk). A portion of the individuals receive outpatient care, which may include treatment with an anti-viral. More complicated cases receive inpatient care. It was assumed that individuals had the same probability of being admitted to hospital whether or not they received outpatient care. While individuals do die from influenza in the community,[8] we conservatively assumed that death occurs only in those who are hospitalised.

Figure S1. Transmission model structure

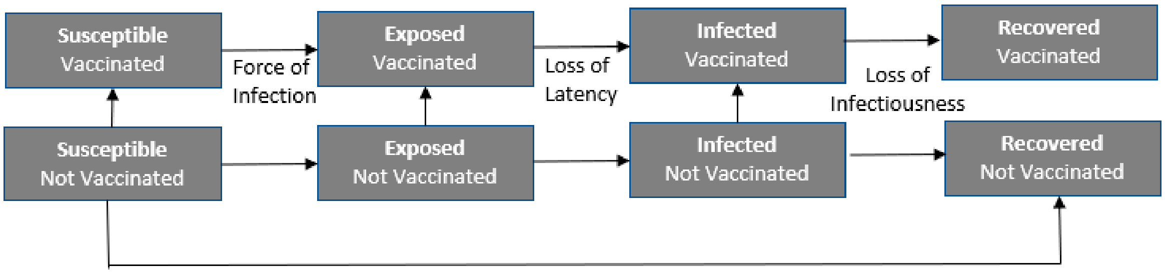

Figure S2. Resource use model structure

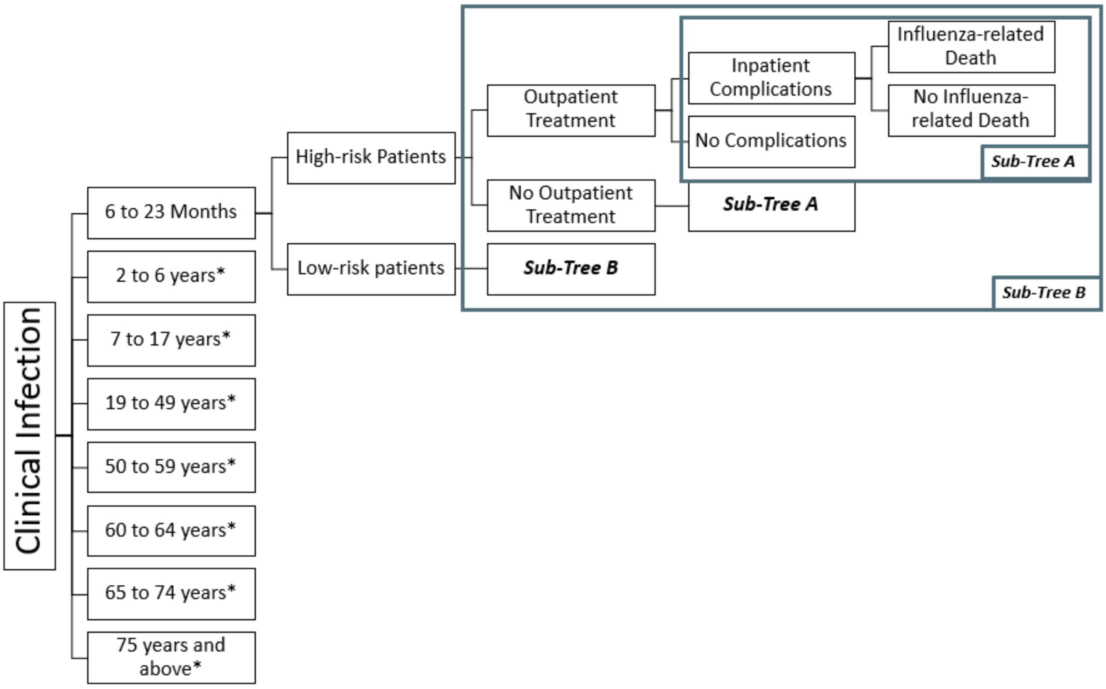

* The tree structure is repeated for all age groups, but this is not shown in the illustration.

2. Mathematical Equations for the Dynamic Model

Differential Equations

$S^{N}_{t+1,ik} = S^{N}_{t,ik} - \lambda_{t,i}S^{N}_{t,ik} - \mu_{t,ik}S^{N}_{t,ik}$<br>$E^{1N}_{t+1,ik} = E^{1N}_{t,ik} + \lambda_{t,i}S^{N}_{t,ik} - \gamma_1 E^{1N}_{t,ik} - \mu_{t,ik}E^{1N}_{t,ik}$<br>$E^{2N}_{t+1,ik} = E^{2N}_{t,ik} + \gamma_1\left(E^{1N}_{t,ik} - E^{2N}_{t,ik}\right) - \mu_{t,ik}E^{2N}_{t,ik}$<br>$I^{1N}_{t+1,ik} = I^{1N}_{t,ik} + \gamma_1 E^{2N}_{t,ik} - \gamma_2 I^{1N}_{t,ik} - \mu_{t,ik}I^{1N}_{t,ik}$<br>$I^{2N}_{t+1,ik} = I^{2N}_{t,ik} + \gamma_2\left(I^{1N}_{t,ik} - I^{2N}_{t,ik}\right) - \mu_{t,ik}I^{2N}_{t,ik}$<br>$R^{N}_{t+1,ik} = R^{N}_{t,ik} + \gamma_2 I^{2N}_{t,ik} - \mu_{t,ik}R^{N}_{t,ik}$	$S^{V}_{t+1,ik} = S^{V}_{t,ik} - \lambda_{t,i}S^{V}_{tmik} + (1-\alpha_i)\mu_{t,ik}S^{N}_{t,ik}$<br>$E^{1V}_{t+1,ik} = E^{1V}_{t,ik} + \lambda_{t,i}S^{V}_{t,ik} - \gamma_1 E^{1V}_{t,ik} + \mu_{t,ik}E^{1N}_{t,ik}$<br>$E^{2V}_{t+1,ik} = E^{2V}_{t,ik} + \gamma_1\left(E^{1V}_{t,ik} - E^{2V}_{t,ik}\right) + \mu_{t,ik}E^{2N}_{t,ik}$<br>$I^{1V}_{t+i,k} = I^{1V}_{t,ik} + \gamma_1 E^{2V}_{t,ik} - \gamma_2 I^{1V}_{t,ik} + \mu_{t,ik}I^{1N}_{t,ik}$<br>$I^{2V}_{t+1,ik} = I^{2V}_{t,ik} + \gamma_2\left(I^{1V}_{t,ik} - I^{2V}_{t,ik}\right) + \mu_{t,ik}I^{2N}_{t,ik}$<br>$R^{V}_{t+1,ik} = R^{V}_{t,ik} + \gamma_2 I^{2V}_{t,ik} + \mu_{t,ik}R^{N}_{t,ik} + \mu_{t,ik}\alpha_i S^{N}_{t,ik}$
For t=0,<br>$S^{N}_{0,ik}$ = Initial number of susceptibles<br>$I^{1N}_{0,ik}$= Initial number of infectious individuals<br>$E^{1N}_{0,ik} = E^{2N}_{0,ik} = I^{2N}_{0,ik} = R^{N}_{0,ik} = 0$	For t=0,<br>$S^{V}_{0,ik} = E^{1N}_{0,ik} = E^{2N}_{0,ik} = I^{1V}_{0,ik} = I^{2N}_{0,ik} = R^{N}_{0,ik} = 0$

where

S^{N}_{ik} and S^{V}_{ik} represent the number of susceptibles of age group i and risk group k with different vaccine history.
The susceptibles indexed by N are naive and the ones indexed by V have received the vaccine.

E^{1X}_{ik} and E^{2X}_{ik} represent exposed, but not yet infectious individuals with vaccine history X.

I^{1X}_{ik} and I^{2X}_{ik} represent infectious individuals with vaccine history X.

R^{X}_{ik} represent immune individuals of age class i and risk group k with vaccine history X

α_i is the proportion of the vaccinees become protected (we assume full protection), while the rest $1 - \alpha_i$ remain fully susceptible. The vaccine efficacy α_i depends on age group and the degree of match between the strain in the vaccine and the circulating strain in that year.

γ_1, γ_2 represent the rate of loss of latency (γ_1) and infectiousness (γ_2) in both groups. Hence, the average latent period is $2/\gamma_1$, and the average infectious period is $2/\gamma_2$.

Force of Infection

$$\lambda_{t,i}^{*} = q\sigma_i \sum_{j=0}^{100} \sum_{k=1}^{2} \sum_{X=\{N,V\}} c_{ij} \left(\frac{I_{t,jk}^{1X} + I_{t,jk}^{2X}}{N_j} \right)$$

$\lambda_{t,i}^{*}$ is the age-group specific force of infection

q is the transmissibility parameter,

σ_i is the susceptibility of age group i

c_{ij} is the rate at which individuals in age group i make contact with those in age group j

I_{ik}^{1X} and I_{ik}^{2X} represent infectious individuals with vaccine history X.

N_j is the total number of people in age group j

3. Dynamic Model Calibration

During the 2010/11 to 2018/19 influenza seasons, Public Health England reported 3 of 9 seasons where the proportion of influenza infections caused by type B was above 20%. We therefore created an A only scenario and an A&B scenario to represent the seasons with higher versus lower rates of B infections. We parameterized these two scenarios using calibration as described presently.

The calibration target was the age-specific cumulative incidence of clinical infection across one influenza season in an unvaccinated population. The A only scenario was calibrated to the median estimate of incidence of clinical influenza in an unvaccinated population from Baguelin 2012[6], which represents an overall cumulative incidence of 8.4% in the UK population for one season. The A&B scenario was calibrated so that the overall incidence of clinical infection in an unvaccinated population was similar to the upper 95% confidence interval from Baguelin 2012.[6] The proportion of infections attributed to B was assumed to be 55%, which was the average of the 3 "high B" seasons in the UK (in 2010/11, 2012/13, 2017/18, the proportion of B was 63.5%, 48.5% and 52.9% respectively). The overall cumulative incidence of influenza A was 6.1% and influenza B was 7.5% in an unvaccinated population for the calibration of this scenario.

Several parameters were fixed based on the literature or previous cost-effectiveness analyses. The numbers of people in each of the 15 age-groups in the dynamic model were based on 2019 data from the Office for National Statistics[9] and separated into low risk and at-risk groups based on previous publications.[10-12] The average latent period was 0.77 days and the average duration of infectiousness was 1.59 days.[6] It was assumed that 100 infections in each risk group seeded or started the influenza infection.

The transmissibility, susceptibility to infection, and percent of infected cases with clinical symptoms parameters were varied during the calibration process based on distributions informed by previous publications by Baguelin and colleagues.[6,12] Transmissibility of infection was allowed to range between 0 and 0.3 across a uniform distribution for the calibration process. The probability of clinical symptoms was allowed to vary between 0 and 0.58 across a uniform distribution. The characteristics of the beta distributions used for age-specific susceptibility inputs are shown in Table S1. One thousand combinations of these inputs were created using Latin hypercube sampling.[13] The model was run with each parameter set and the R_0, or basic reproductive number, was calculated using the simulation method described by Vynnycky and White.[14] The effective reproductive number, R_e, was defined in this study, similar to Baguelin 2013[12], as the reproductive number at the start of the influenza season considering pre-existing susceptibility and was also calculated using the simulation method described by Vynnycky and White.[14]. Parameter sets that produced epidemics with R_e between 1.20 and 1.30 (as in Baguelin 2013[12]) were considered viable. The viable set that produced the best goodness of fit (using a least square measure) to the calibration targets was chosen.[13] The final calibrated input parameters are shown in Table S2. The incidence of clinical infection in the unvaccinated population for each scenario is shown in Figure S3 and Figure S4.

Table S1. Characteristics of the distribution of age-specific susceptibility distribution used in the calibration.

Age Group	Mean	Median	Lower 95% CI	Upper 95% CI	Distributio n
0-23 months	0.69	0.66	0.53	0.88	Beta
2-6 years	0.69	0.66	0.53	0.88	Beta
7-17 years	0.76	0.77	0.54	0.89	Beta
18-24 years	0.72	0.74	0.51	0.88	Beta
25-29 years	0.73	0.74	0.55	0.89	Beta
30-34 years	0.73	0.74	0.55	0.89	Beta
35-39 years	0.73	0.74	0.55	0.89	Beta
40-44 years	0.73	0.74	0.55	0.89	Beta
45-49 years	0.68	0.68	0.52	0.88	Beta
50-54 years	0.68	0.68	0.52	0.88	Beta
55-59 years	0.68	0.68	0.52	0.88	Beta
60-64 years	0.68	0.68	0.52	0.88	Beta
65-69 years	0.7	0.7	0.52	0.89	Beta
70-74 years	0.7	0.7	0.52	0.89	Beta
75+ years	0.7	0.7	0.52	0.89	Beta

Table S2. Final dynamic model inputs, by scenario

Parameter	A Only Scenario	Low A; High B Scenario (A parameters)	Low A; High B Scenario (B parameters)
**Transmissibility**	0.086	0.086	0.089
**Percent of infection with clinical symptoms**	0.316	0.277	0.296
**Susceptibility to infection**			
0-1 years	0.735	0.862	0.709
2-6 years	0.727	0.71	0.718
7-17 years	0.783	0.831	0.7
18-24 years	0.754	0.727	0.784
25-29 years	0.822	0.65	0.649
30-34 years	0.843	0.692	0.607
35-39 years	0.715	0.481	0.696
40-44 years	0.61	0.83	0.751
45-49 years	0.601	0.532	0.645
50-54 years	0.588	0.692	0.714
55-59 years	0.604	0.528	0.553
60-64 years	0.677	0.618	0.718
65-69 years	0.775	0.687	0.656
70-74 years	0.579	0.734	0.645
75+ years	0.79	0.69	0.779

Figure S3. Incidence of clinical infection in an unvaccinated population (A only scenario)

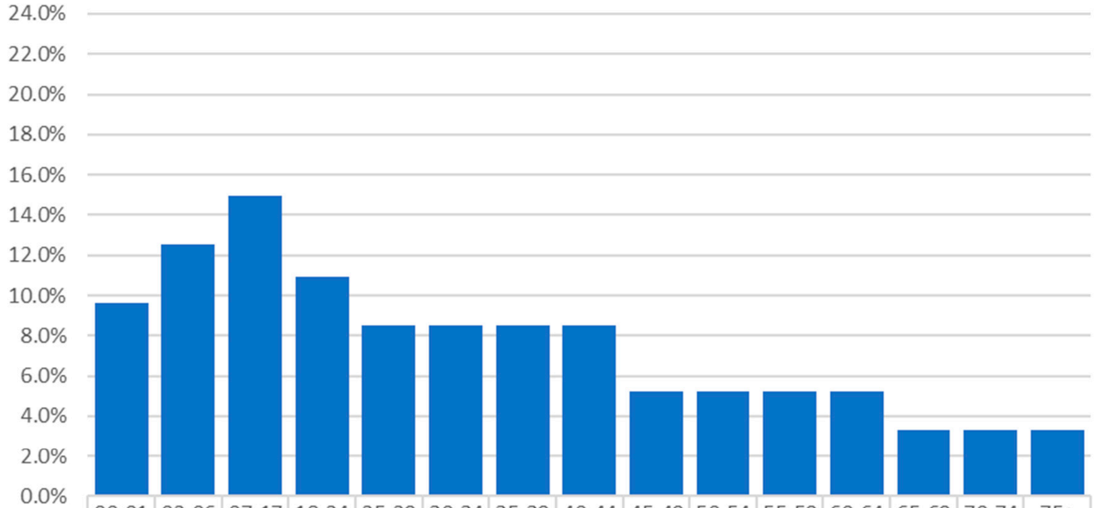

	00-01	02-06	07-17	18-24	25-29	30-34	35-39	40-44	45-49	50-54	55-59	60-64	65-69	70-74	75+
Influenza B	0.0%	0.0%	0.0%	0.0%	0.0%	0.0%	0.0%	0.0%	0.0%	0.0%	0.0%	0.0%	0.0%	0.0%	0.0%
Influenza A	9.7%	12.5%	15.0%	10.9%	8.5%	8.5%	8.5%	8.5%	5.2%	5.2%	5.2%	5.2%	3.3%	3.3%	3.3%

Influenza A Influenza B

Figure S4. Incidence of clinical infection in an unvaccinated population (Low A / High B Scenario)

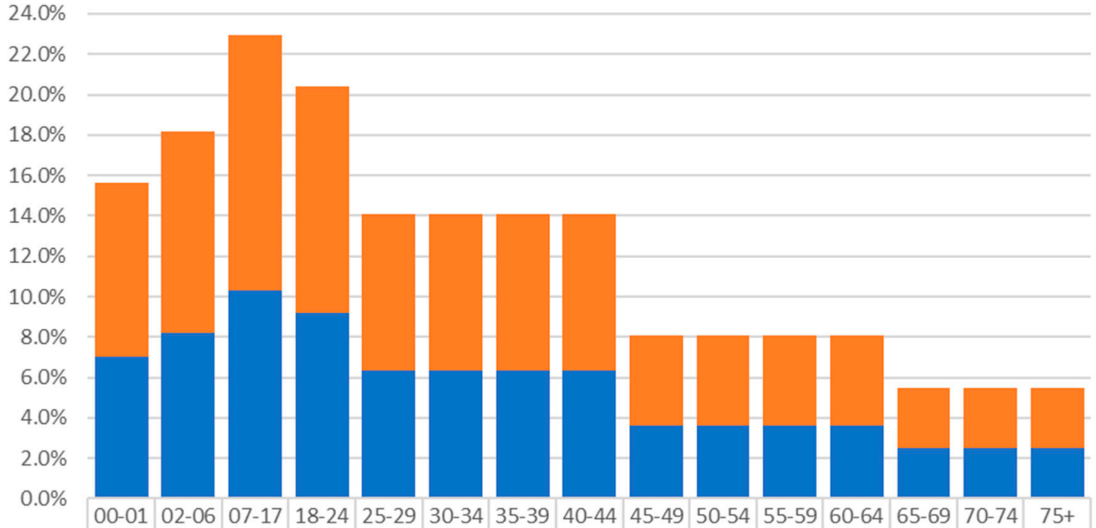

	00-01	02-06	07-17	18-24	25-29	30-34	35-39	40-44	45-49	50-54	55-59	60-64	65-69	70-74	75+
Influenza B	8.6%	10.0%	12.6%	11.2%	7.8%	7.8%	7.8%	7.8%	4.5%	4.5%	4.5%	4.5%	3.0%	3.0%	3.0%
Influenza A	7.0%	8.2%	10.3%	9.2%	6.3%	6.3%	6.3%	6.3%	3.6%	3.6%	3.6%	3.6%	2.5%	2.5%	2.5%

Influenza A Influenza B

4. Calibration of Hospitalization Rates

The number of hospitalizations expected in the population on an annual basis, by age and risk group, was determined based upon an analysis conducted by Cromer and colleagues[15]. This analysis was conducted using the Hospital Episode Statistics database for the 2000/1 to 2007/8 seasons. Hospital admissions for acute respiratory illness were linked using a regression equation to weekly microbiological laboratory reports from Public Health England, where the organisms in a sample of reported respiratory illness have been typed, in order to attribute the admission to a particular pathogen. The overall rates, rather than the type-specific rates, of hospitalization due to influenza stratified by age and risk groups were used to derive the base-case inputs. The conditional probability of influenza given infection was then calibrated so that the average expected number of hospital admissions due to influenza over the 9 seasons time horizon used for the calibration matched these expected rates of hospitalization in the population under a vaccination strategy based on 2019/20 influenza season vaccination recommendations.[16,17]

5. Vaccine Effectiveness

The data used for the effectiveness of the cellular-based quadrivalent influenza vaccine (QIVc) were described in the manuscript text.

For the other vaccines, effectiveness depended upon the type of influenza strains circulating each season of the analysis. The proportion of infections seen in the 10 years analysed were based on data from the past 10 seasons in the UK. [18-27] An A&B season occurred in only 3 years while the proportion of A infections due to H1N1 and H3N2 changed annually as shown in Table S3.

For quadrivalent live attenuated influenza vaccine (QLAIV), data from a systematic review of RCTs and quasi-RCTs by Tricco and colleagues was used to estimate the strain-specific effectiveness for those 18 years and under (Table S4).[28]The age and strain-specific effectiveness of the egg-based quadrivalent influenza vaccine (QIVe) was estimated based on a systematic review of test-negative design studies of influenza vaccination conducted by Belongia and colleagues.[29] Several assumptions were made in order to use these data. First, Belongia did not limit the review to a particular type of vaccine, so it was assumed that the presented data applied to inactivated egg-grown vaccines as this would have been the most common vaccine used during the study period. Unlike other reviews,[28,30] adjustments were made to the B efficacy using data from Tricco and colleagues[28] because the vaccines reviewed by Belongia would have been primarily trivalent formulations. On average, trivalent formulations are less

effective than quadrivalent formulations because they contain one B strain, which may or may not have matched the local circulating strain.

For aQIV, the effectiveness was calculated as being relative to QIVe for each season using the data for ages 65 years and above in Table S4. The relative vaccine effectiveness (rVE) of aQIV compared to aQIV was assumed to be 0.20 based on previously published cost-effectiveness analyses for the UK.[31]

Table S3. Characteristics of the 10 influenza seasons used in the analysis

Year	Scenario	Proportion of A strain	
		A/H1N1	A/H3N2
1	Low A/ High B	97.1%	2.9%
2	A Only	0.7%	99.3%
3	Low A/ High B	20.8%	79.2%
4	A Only	78.3%	21.7%
5	A Only	16.4%	83.6%
6	A Only	98.2%	1.8%
7	A Only	1.0%	99.0%
8	Low A/ High B	31.4%	68.6%
9	A Only	80.2%	19.8%
10	A Only	11.0%	89.0%

Table S4. Absolute effectiveness inputs for QIVe and QLAIV by age and viral type

Vaccine	Age Group	QIVe	QLAIV
A/H1N1	Under 18	0.69	0.78
	18 to 64	NA	NA
	65 and above	0.62	NA
A/H3N2	Under 18	0.43	0.78
	18 to 64	NA	NA
	65 and above	0.24	NA
B	Under 18	0.70	0.77
	18 to 64	NA	NA
	65 and above	0.79	NA
	18 to 64	NA	NA
	65 and above	NA	NA
Source		Belongia 2016[29]	Tricco 2013[28]

NA – Not applicable; QIVe - Quadrivalent inactivated (egg-grown); QLAIV Quadrivalent live attenuated nasal spray

6. Probabilistic Sensitivity Analyses

A probabilistic sensitivity analysis was conducted with each model with a Monte Carlo simulation of 1,000 runs. Standard errors for the inputs were calculated based on the 95% confidence interval or the range (minimum or maximum) provided in the source study described in the manuscript text.

Table S5. Probabilistic sensitivity analysis base case values and standard errors

Parameter Description	Base Case Value	Standard Error	Distribution
% of patients with an infection seeking a medical care visit, at-risk, 6-23 months	10.0%	2.2%	Beta
% of patients with an infection seeking a medical care visit, at-risk, 2-6 yrs	10.0%	2.2%	Beta
% of patients with an infection seeking a medical care visit, at-risk, 7-17 yrs	10.0%	2.2%	Beta
% of patients with an infection seeking a medical care visit, at-risk, 18-49 yrs	10.0%	2.2%	Beta
% of patients with an infection seeking a medical care visit, at-risk, 50-59 yrs	10.0%	2.2%	Beta
% of patients with an infection seeking a medical care visit, at-risk, 60-64 yrs	10.0%	2.2%	Beta
% of patients with an infection seeking a medical care visit, at-risk, 65-74 yrs	10.0%	2.2%	Beta
% of patients with an infection seeking a medical care visit, at-risk, 75 + yrs	10.0%	2.2%	Beta
% of patients with an infection seeking a medical care visit, low risk, 6-23 months	10.0%	2.2%	Beta
% of patients with an infection seeking a medical care visit, low risk, 2-6 yrs	10.0%	2.2%	Beta
% of patients with an infection seeking a medical care visit, low risk, 7-17 yrs	10.0%	2.2%	Beta
% of patients with an infection seeking a medical care visit, low risk, 18-49 yrs	10.0%	2.2%	Beta
% of patients with an infection seeking a medical care visit, low risk, 50-59 yrs	10.0%	2.2%	Beta
% of patients with an infection seeking a medical care visit, low risk, 60-64 yrs	10.0%	2.2%	Beta
% of patients with an infection seeking a medical care visit, low risk, 65-74 yrs	10.0%	2.2%	Beta
% of patients with an infection seeking a medical care visit, low risk, 75 + yrs	10.0%	2.2%	Beta
Overall Inpatient Complications, at-risk, 6-23 months	1.610%	0.058%	Beta
Overall Inpatient Complications, at-risk, 2-6 yrs	1.460%	0.050%	Beta
Overall Inpatient Complications, at-risk, 7-17 yrs	0.560%	0.013%	Beta
Overall Inpatient Complications, at-risk, 18-49 yrs	0.760%	0.011%	Beta

Parameter Description	Base Case Value	Standard Error	Distribution
Overall Inpatient Complications, at-risk, 50-59 yrs	1.960%	0.040%	Beta
Overall Inpatient Complications, at-risk, 60-64 yrs	1.960%	0.040%	Beta
Overall Inpatient Complications, at-risk, 65-74 yrs	4.150%	0.173%	Beta
Overall Inpatient Complications, at-risk, 75 + yrs	4.670%	0.195%	Beta
Overall Inpatient Complications low risk, 6-23 months	1.820%	0.026%	Beta
Overall Inpatient Complications low risk, 2-6 yrs	1.110%	0.017%	Beta
Overall Inpatient Complications low risk, 7-17 yrs	0.080%	0.003%	Beta
Overall Inpatient Complications low risk, 18-49 yrs	0.120%	0.002%	Beta
Overall Inpatient Complications low risk, 50-59 yrs	0.310%	0.003%	Beta
Overall Inpatient Complications low risk, 60-64 yrs	0.310%	0.003%	Beta
Overall Inpatient Complications low risk, 65-74 yrs	2.270%	0.025%	Beta
Overall Inpatient Complications low risk, 75 + yrs	2.560%	0.028%	Beta
Influenza-related mortality rate, at-risk, 6-23 months	1.75%	0.003%	Beta
Influenza-related mortality rate, at-risk, 2-6 yrs	1.75%	0.003%	Beta
Influenza-related mortality rate, at-risk, 7-17 yrs	2.44%	0.007%	Beta
Influenza-related mortality rate, at-risk, 18-49 yrs	4.00%	0.018%	Beta
Influenza-related mortality rate, at-risk, 50-59 yrs	4.00%	0.018%	Beta
Influenza-related mortality rate, at-risk, 60-64 yrs	4.00%	0.018%	Beta
Influenza-related mortality rate, at-risk, 65-74 yrs	42.85%	0.332%	Beta
Influenza-related mortality rate, at-risk, 75 + yrs	42.85%	0.332%	Beta
Influenza-related mortality rate, low risk, 6-23 months	0.04%	0.077%	Beta
Influenza-related mortality rate, low risk, 2-6 yrs	0.04%	0.077%	Beta
Influenza-related mortality rate, low risk, 7-17 yrs	0.07%	0.098%	Beta
Influenza-related mortality rate, low risk, 18-49 yrs	0.61%	0.153%	Beta
Influenza-related mortality rate, low risk, 50-59 yrs	0.61%	0.153%	Beta
Influenza-related mortality rate, low risk, 60-64 yrs	0.61%	0.153%	Beta
Influenza-related mortality rate, low risk, 65-74 yrs	18.53%	2.092%	Beta
Influenza-related mortality rate, low risk, 75 + yrs	18.53%	2.092%	Beta
QALYs lost for infections with complications, at-risk, 6-23 months	0.01800	0.00180	Beta
QALYs lost for infections with complications, at-risk, 2-6 yrs	0.01800	0.00180	Beta
QALYs lost for infections with complications, at-risk, 7-17 yrs	0.01800	0.00180	Beta
QALYs lost for infections with complications, at-risk, 18-49 yrs	0.01800	0.00180	Beta

Parameter Description	Base Case Value	Standard Error	Distribution
QALYs lost for infections with complications, at-risk, 50-59 yrs	0.01800	0.00180	Beta
QALYs lost for infections with complications, at-risk, 60-64 yrs	0.01800	0.00180	Beta
QALYs lost for infections with complications, at-risk, 65-74 yrs	0.01800	0.00180	Beta
QALYs lost for infections with complications, at-risk, 75 + yrs	0.01800	0.00180	Beta
QALYs lost for infections with complications, low risk, 6-23 months	0.01800	0.00180	Beta
QALYs lost for infections with complications, low risk, 2-6 yrs	0.01800	0.00180	Beta
QALYs lost for infections with complications, low risk, 7-17 yrs	0.01800	0.00180	Beta
QALYs lost for infections with complications, low risk, 18-49 yrs	0.01800	0.00180	Beta
QALYs lost for infections with complications, low risk, 50-59 yrs	0.01800	0.00180	Beta
QALYs lost for infections with complications, low risk, 60-64 yrs	0.01800	0.00180	Beta
QALYs lost for infections with complications, low risk, 65-74 yrs	0.01800	0.00180	Beta
QALYs lost for infections with complications, low risk, 75 + yrs	0.01800	0.00180	Beta
QALYs lost for infections, no complications, 6-23 months	0.0075	0.0073	Beta
QALYs lost for infections, no complications, 2-6 yrs	0.0075	0.0073	Beta
QALYs lost for infections, no complications, 7-17 yrs	0.0075	0.0073	Beta
QALYs lost for infections, no complications, 18-49 yrs	0.0075	0.0073	Beta
QALYs lost for infections, no complications, 50-59 yrs	0.0075	0.0073	Beta
QALYs lost for infections, no complications, 60-64 yrs	0.0075	0.0073	Beta
QALYs lost for infections, no complications, 65-74 yrs	0.0075	0.0073	Beta
QALYs lost for infections, no complications, 75 + yrs	0.0075	0.0073	Beta

7. Additional Results Tables and Figures

Table S6. Base case results: the average annual change in clinical cases when low-risk individuals ages 50 to 64 years old are vaccinated, by coverage level and age group.

	Number with Current Strategy (0% low-risk)	Change with 40% (50 to 64 years)	Change with 50% (50 to 64 years)	Change with 60% (50 to 64 years)
**Vaccinations**				
50 - 64 year old	1,142,585	4,198,394	5,280,907	6,565,605
Other age groups	14,997,933	0	0	0
**Total**	**16,140,518**	**4,198,394**	**5,280,907**	**6,565,605**
**Clinical Infection**				
50 - 64 year old	338,822	-123,703	-151,257	-181,775
Other age groups	2,276,756	-421,015	-530,780	-662,502
**Total**	**2,615,577**	**-544,718**	**-682,036**	**-844,277**
**Hospitalizations**				
50 - 64 year old	3,207	-969	-1,204	-1,530
Other age groups	18,942	-3,660	-4,603	-5,727
**Total**	**22,148**	**-4,629**	**-5,807**	**-7,257**
**Deaths**				
50 - 64 year old	74	-17	-22	-31
Other age groups	2,761	-571	-715	-885
**Total**	**2,834**	**-588**	**-737**	**-916**

Table S7. Base case results: the average annual discounted costs and quality-adjusted life-years with different level of coverage of low-risk individuals aged 50 to 64 years old, by age group.

	Current Strategy (0% Coverage of Low-Risk)	40% Coverage of Low-Risk	50% Coverage of Low-Risk	60% Coverage of Low-Risk
**Cost of Vaccinations**				
50 - 64 year old	£9,774,056	£45,688,532	£54,948,708	£65,938,450
Other age groups	£170,709,774	£170,709,774	£170,709,774	£170,709,774
**Cost of Vaccine Administration**				
50 - 64 year old	£9,892,053	£46,240,104	£55,612,073	£66,734,487
Other age groups	£129,348,844	£129,348,844	£129,348,844	£129,348,844
**Cost of Medical Care Visits**				
50 - 64 year old	£3,653,038	£2,316,352	£2,018,942	£1,689,281
Other age groups	£19,128,182	£15,534,944	£14,601,311	£13,482,767
**Cost of Hospitalisations**				
50 - 64 year old	£6,785,662	£4,725,934	£4,226,991	£3,536,902
Other age groups	£64,611,357	£51,603,280	£48,290,064	£44,356,671
**Total NHS Costs**				
50 - 64 year old	£30,104,809	£98,970,921	£116,806,714	£137,899,120
Other age groups	£383,798,157	£367,196,842	£362,949,993	£357,898,056
**All age groups**	**£413,902,966**	**£466,167,764**	**£479,756,707**	**£495,797,175**
**Productivity**				
50 - 64 year old	£97,229,817	£61,611,487	£53,690,409	£44,923,611
Other age groups	£441,436,376	£357,359,117	£335,580,845	£309,531,855
**Total Societal Costs**				
50 - 64 year old	£127,334,626	£160,582,408	£170,497,123	£182,822,730
Other age groups	£825,234,533	£724,555,959	£698,530,838	£667,429,910
**All age groups**	**£952,569,159**	**£885,138,367**	**£869,027,961**	**£850,252,641**
**QALYs**				
50 - 64 year old	9,285,196	9,286,271	9,286,525	9,286,854
Other age groups	42,129,290	42,136,935	42,138,890	42,141,215
**All age groups**	**51,414,486**	**51,423,206**	**51,425,415**	**51,428,069**

NHS – National Health Service; QALY – Quality-adjusted life-years

Table S8. The impact of days lost per case of influenza on productivity costs and the societal costs per quality adjusted life-year gained.

Sensitivity analyses		1	Base Case	2	3	4	5
**Time Lost from Work Due to Vaccination (Hours)**		0	0	0	1	1	1
**Time Lost From Work Due to Influenza (Days)**		1.2	4.0	6.8	1.2	4.0	6.8
**Average Annual Discounted Productivity Costs**							
**Current Strategy**	**50 to 64 year olds**	£29,168,945	£97,229,817	£165,290,689	£41,698,617	£109,759,489	£177,820,361
	**Other age groups**	£132,430,913	£441,436,376	£750,441,839	£165,081,918	£474,087,381	£783,092,844
	**Total**	**£161,599,858**	**£538,666,193**	**£915,732,528**	**£206,780,534**	**£583,846,869**	**£960,913,204**
**50% Coverage of Low-Risk**	**50 to 64 year olds**	£16,107,123	£53,690,409	£91,273,696	£86,547,607	£124,130,894	£161,714,180
	**Other age groups**	£100,674,254	£335,580,845	£570,487,437	£133,325,258	£368,231,850	£603,138,442
	**Total**	**£116,781,376**	**£389,271,255**	**£661,761,133**	**£219,872,866**	**£492,362,744**	**£764,852,622**
**Incremental (50% - Current)**	**50 to 64 year olds**	-£13,061,822	-£43,539,408	-£74,016,993	£44,848,991	£14,371,405	-£16,106,180
	**Other age groups**	-£31,756,659	-£105,855,530	-£179,954,402	-£31,756,659	-£105,855,530	-£179,954,402
	**Total**	**-£44,818,481**	**-£149,394,938**	**-£253,971,395**	**£13,092,332**	**-£91,484,125**	**-£196,060,582**
**Total Average Annual Discounted Societal Costs**							
Current Strategy		£575,502,824	£952,569,159	£1,329,635,494	£620,683,500	£997,749,835	£1,374,816,170
50% Coverage of Low-Risk		£596,538,083	£869,027,961	£1,141,517,840	£699,629,573	£972,119,451	£1,244,609,329
Incremental (50% - Current)		**£21,035,259**	**-£83,541,197**	**-£188,117,654**	**£78,946,072**	**-£25,630,384**	**-£130,206,841**

Figure S5. The cost-effectiveness acceptability curves for probabilistic sensitivity analyses with vaccine effectiveness set to 26.9% (Panel A), 63.9% (Panel B), and 82.2% (Panel C).

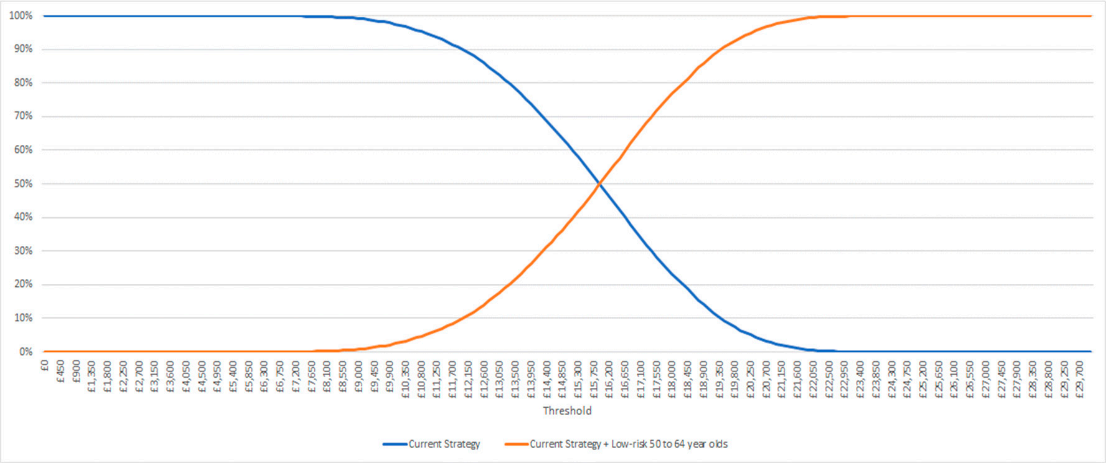

A

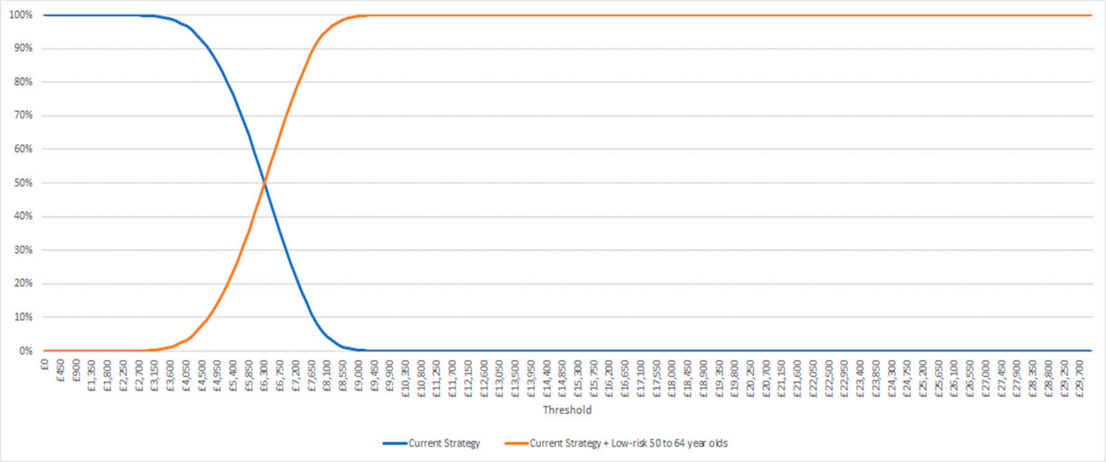

B

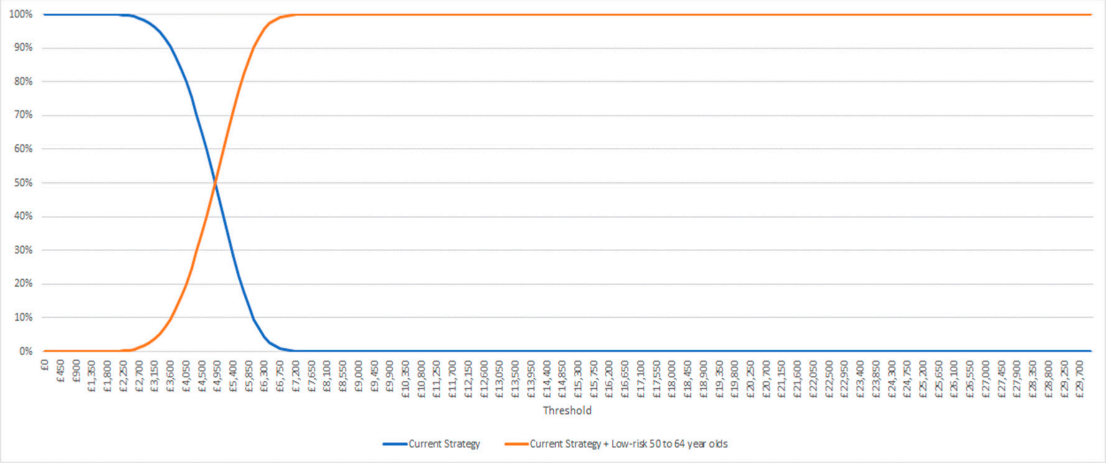

C

8. References

1. Vynnycky, E.; Pitman, R.; Siddiqui, R.; Gay, N.; Edmunds, W.J. Estimating the impact of childhood influenza vaccination programmes in England and Wales. *Vaccine* **2008**, *26*, 5321-5330, doi:10.1016/j.vaccine.2008.06.101.
2. Pitman, R.J.; White, L.J.; Sculpher, M. Estimating the clinical impact of introducing paediatric influenza vaccination in England and Wales. *Vaccine* **2012**, *30*, 1208-1224, doi:10.1016/j.vaccine.2011.11.106.
3. Pitman, R.J.; Nagy, L.D.; Sculpher, M.J. Cost-effectiveness of childhood influenza vaccination in England and Wales: Results from a dynamic transmission model. *Vaccine* **2013**, *31*, 927-942, doi:10.1016/j.vaccine.2012.12.010.
4. Thommes, E.W.; Ismaila, A.; Chit, A.; Meier, G.; Bauch, C.T. Cost-effectiveness evaluation of quadrivalent influenza vaccines for seasonal influenza prevention: a dynamic modeling study of Canada and the United Kingdom. *BMC Infect Dis* **2015**, *15*, 465, doi:10.1186/s12879-015-1193-4.
5. Mossong, J.; Hens, N.; Jit, M.; Beutels, P.; Auranen, K.; Mikolajczyk, R.; Massari, M.; Salmaso, S.; Tomba, G.S.; Wallinga, J.; et al. Social contacts and mixing patterns relevant to the spread of infectious diseases. *PLoS Med* **2008**, *5*, e74, doi:10.1371/journal.pmed.0050074.
6. Baguelin, M.; Jit, M.; Miller, E.; Edmunds, W.J. Health and economic impact of the seasonal influenza vaccination programme in England. *Vaccine* **2012**, *30*, 3459-3462, doi:10.1016/j.vaccine.2012.03.019.
7. Keeling, M.J.; Rohani, P. *Modeling Infectious Diseases in Humans and Animals*; Princeton University Press: Priceton and Oxford, 2008.
8. Johnson, B.F.; Wilson, L.E.; Ellis, J.; Elliot, A.J.; Barclay, W.S.; Pebody, R.G.; McMenamin, J.; Fleming, D.M.; Zambon, M.C. Fatal cases of influenza a in childhood. *PLoS One* **2009**, *4*, e7671, doi:10.1371/journal.pone.0007671.
9. Office for National Statistics. 2016-based National Population Projections. Available Online at: https://www.ons.gov.uk/peoplepopulationandcommunity/populationandmigration/populationprojections. Available online: (accessed on April 16, 2019).

10. Thorrington, D.; van Leeuwen, E.; Ramsay, M.; Pebody, R.; Baguelin, M. Cost-effectiveness analysis of quadrivalent seasonal influenza vaccines in England. *BMC Med* **2017**, *15*, 166, doi:10.1186/s12916-017-0932-3.
11. Baguelin, M.; Camacho, A.; Flasche, S.; Edmunds, W.J. Extending the elderly- and risk-group programme of vaccination against seasonal influenza in England and Wales: a cost-effectiveness study. *BMC Med* **2015**, *13*, 236, doi:10.1186/s12916-015-0452-y.
12. Baguelin, M.; Flasche, S.; Camacho, A.; Demiris, N.; Miller, E.; Edmunds, W.J. Assessing optimal target populations for influenza vaccination programmes: an evidence synthesis and modelling study. *PLoS Med* **2013**, *10*, e1001527, doi:10.1371/journal.pmed.1001527.
13. Vanni, T.; Karnon, J.; Madan, J.; White, R.G.; Edmunds, W.J.; Foss, A.M.; Legood, R. Calibrating models in economic evaluation: a seven-step approach. *Pharmacoeconomics* **2011**, *29*, 35-49, doi:10.2165/11584600-000000000-00000.
14. Vynnycky, E.; White, R.G. *An introduction to infectious disease modelling.*; Oxford University Press Oxford, UK., 2010.
15. Cromer, D.; van Hoek, A.J.; Jit, M.; Edmunds, W.J.; Fleming, D.; Miller, E. The burden of influenza in England by age and clinical risk group: a statistical analysis to inform vaccine policy. *J Infect* **2014**, *68*, 363-371, doi:10.1016/j.jinf.2013.11.013.
16. Public Health England; Department of Health & Social Care; NHS England. The national flu immuisation programme 2019/20. 22 March 2019. Available Online at: https://www.england.nhs.uk/wp-content/uploads/2019/03/annual-national-flu-programme-2019-to-2020-1.pdf.
17. Public Health England. Flu vaccines: 2019 to 2020 flu season (Poster). Available Online at: https://www.gov.uk/government/collections/annual-flu-programme.
18. Health Protection Agency. *Surveillance of influenza and other respiratory viruses in the UK, 2010/11.*; London, UK. , 2011.
19. Health Protection Agency. *Surveillance of influenza and other respiratory pathogens in the UK. October 2011 - April 2012.*; London, UK. , 2012.
20. Public Health England. *Surveillance of influenza and other respiratory viruses, including novel respiratory viruses, in the United Kingdom: Winter 2012/13.*; London, UK. , 2013.
21. Public Health England. *Surveillance of influenza and other respiratory viruses in the United Kingdom: Winter 2013/14.*; London, UK. , 2014.
22. Public Health England. *Surveillance of influenza and other respiratory viruses in the United Kingdom: winter 2014 to 2015.*; 2015.

23. Public Health England. *Surveillance of influenza and other respiratory viruses in the United Kingdom: Winter 2015 to 2016.*; London, UK. , 2016.
24. Public Health England. *Surveillance of influenza and other respiratory viruses in the UK: Winter 2016 to 2017.*; London, UK. , 2017.
25. Public Health England. *Surveillance of influenza and other respiratory viruses in the UK: Winter 2017 to 2018.*; London, UK. , 2018.
26. Public Health England. *Surveillance of influenza and other respiratory viruses in the UK: Winter 2018 to 2019.*; London, UK. , 2019.
27. Public Health England. *Surveillance of influenza and other respiratory viruses in the UK: Winter 2019 to 2020.*; London, United Kingdom, 2020.
28. Tricco, A.C.; Chit, A.; Soobiah, C.; Hallett, D.; Meier, G.; Chen, M.H.; Tashkandi, M.; Bauch, C.T.; Loeb, M. Comparing influenza vaccine efficacy against mismatched and matched strains: a systematic review and meta-analysis. *BMC Med* **2013**, *11*, 153, doi:10.1186/1741-7015-11-153.
29. Belongia, E.A.; Simpson, M.D.; King, J.P.; Sundaram, M.E.; Kelley, N.S.; Osterholm, M.T.; McLean, H.Q. Variable influenza vaccine effectiveness by subtype: a systematic review and meta-analysis of test-negative design studies. *Lancet Infect Dis* **2016**, *16*, 942-951, doi:10.1016/S1473-3099(16)00129-8.
30. DiazGranados, C.A.; Denis, M.; Plotkin, S. Seasonal influenza vaccine efficacy and its determinants in children and non-elderly adults: a systematic review with meta-analyses of controlled trials. *Vaccine* **2012**, *31*, 49-57, doi:10.1016/j.vaccine.2012.10.084.
31. Thorrington, D.; van Leeuwen, E.; Ramsay, M.; Pebody, R.; Baguelin, M. Assessing optimal use of the standard dose adjuvanted trivalent seasonal influenza vaccine in the elderly. *Vaccine* **2019**, *37*, 2051-2056, doi:10.1016/j.vaccine.2019.03.002.
